# Retraction: Hydrogen Sulfide Attenuated Tumor Necrosis Factor-α-Induced Inflammatory Signaling and Dysfunction in Vascular Endothelial Cells

**DOI:** 10.1371/journal.pone.0311557

**Published:** 2024-10-01

**Authors:** The PLOS ONE Editors

After this article [1] was published, concerns were raised about Figs 3–7.

Specifically, when color levels are adjusted to visualize the backgrounds, the following concerns were identified:

- In Fig 3A, there appear to be vertical discontinuities in the background of the:
  ○ ICAM-1 panel, between lanes 3 and 4.
  ○ VCAM-1 panel, between lanes 1 and 2.
  ○ Tubulin panel, between lanes 1/2 and 2/3.
- In the Fig 4B HO-1 panel, there appears to be a vertical discontinuity in the background between lanes 2 and 3.
- In Fig 5A, similarities were noted between the Control and TNF-a+NAC panels.
- In the Fig 6A ERK panel:
  ○ Similarities were noted between bands in lanes 1 and 4.
  ○ The background area around the band in lane 1 appears to be discontinuous with adjacent background areas.
  ○ There appears to be a vertical discontinuity in the background between lanes 2 and 3.
- In Fig 6B, there appear to be vertical discontinuities in the background of the p-p38 panel between lanes 1 and 2, 3 and 4, and 4 and 5.
- In the Fig 6C JNK panel:
  ○ Similarities were noted between bands in lanes 1 and 5.
  ○ There appears to be a vertical discontinuity in the background between lanes 1 and 2.
- In the Fig 7C pp65 panel, there appears to be a vertical discontinuity in the background between lanes 1 and 2.
- In the Fig 7D p65 (C) panel, there appears to be a vertical discontinuity in the background between lanes 2 and 3.

The authors did not respond to correspondence concerning these issues.

In light of the nature and extent of the above concerns, the *PLOS ONE* Editors retract this article.

All authors either did not respond directly or could not be reached.
